# Validation of the Arab Mental Health Continuum Short Form (AMHC-SF) in Oman: Psychometric properties and factorial structure

**DOI:** 10.1371/journal.pone.0352184

**Published:** 2026-07-21

**Authors:** Sharif Alsoudi, Amina Benkouider, Huda Al-Manjiya

**Affiliations:** 1 Department of Psychology, Arts & Humanities College, A’Sharqiyah University, Ibra, Oman; 2 Director of Al-Thiqa Rehabilitation Center, Ministry of Social Development, Ibra, Oman; Universiti Sains Malaysia, MALAYSIA

## Abstract

**Background:**

The Mental Health Continuum–Short Form (MHC–SF) is a widely used instrument designed to assess three dimensions of positive mental health: emotional, social, and psychological well-being. However, there is limited evidence regarding its validity within Arab populations.

**Method:**

This study aimed to validate the Arabic version of the MHC–SF (AMHC–SF) among a community-based sample in Oman. A total of 1,101 participants aged 18–65 years (M = 31.49, SD = 9.69) completed the questionnaire through an online survey. Confirmatory factor analyses (CFA) were performed to examine factorial validity, while internal consistency reliability was assessed using Cronbach’s alpha and McDonald’s omega coefficients. Discriminant validity was examined through correlations with anxiety scales.

**Results:**

Confirmatory factor analysis supported the superiority of the bifactor model over the original three-factor structure, demonstrating excellent model fit (CFI = 0.99, TLI = 0.98, RMSEA = 0.032) and substantial general factor loadings (0.64–0.76). Reliability estimates for the total scale and subscales were excellent, with Cronbach’s alpha and McDonald’s omega coefficients ranging from 0.86 to 0.95. The AMHC-SF also showed apparent discriminant validity, as higher well-being scores were associated with lower anxiety levels.

**Conclusion:**

The findings support the factorial validity, reliability, and discriminant validity of the AMHC–SF, confirming its cross-cultural applicability within the Arab context. The scale represents a brief, psychometrically sound instrument for assessing positive mental health among adults in Oman and other Arabic-speaking populations.

## Introduction

There are growing global concerns about the prevalence and underlying causes of mental illness [1]. The prevalence of mental disorders has increased substantially over recent decades [2]. Although this escalation has stimulated greater attention to research, policy, diagnosis, and treatment, it has often overshadowed the study of positive mental health and psychological well-being [3]. Such neglect has limited efforts to counter the increasing decline in mental health across the lifespan [4,5]. Individuals at different developmental stages face unique challenges, including inadequate care and attachment in childhood [6], academic stress during adolescence [7], occupational and financial pressures in young adulthood [8], and deteriorating physical health and social isolation in later life [9]. These variations underscore the importance of promoting well-being across developmental contexts. Importantly, mental health should not be viewed solely as the absence of mental illness but also as the presence of positive functioning and flourishing [1].

Within this perspective, the study of psychological well-being has become central to the field of positive psychology, as it assesses mental health beyond the mere absence of pathology. Higher levels of well-being are associated with personal growth, environmental mastery, purpose in life, and self-acceptance [10], as well as adaptive functioning in the face of adversities such as child loss, childhood violence, and work–family stressors [11–14]. Engagement in social and volunteer activities further enhances hedonic, eudaimonic, and social well-being [15,16]. Moreover, well-being therapy has proven effective in fostering growth, self-regulation, and self-acceptance in both clinical and non-clinical populations [10]. Collectively, psychological well-being emerges as a crucial indicator of individuals’ capacity to adapt, thrive, and find meaning in life, highlighting the need for valid and reliable tools to measure it accurately across cultures.

According to Keyes [1], mental health is best conceptualized as a multidimensional construct encompassing emotional, social, and psychological well-being. Based on this framework, the Mental Health Continuum–Short Form (MHC–SF) was developed as a concise yet psychometrically sound measure to assess positive mental health across these dimensions [17]. The MHC–SF has been validated across numerous cultural contexts, demonstrating strong factorial structure and reliability [18,19]. However, research within Arab societies remains limited, and cultural differences in the experience and expression of well-being highlight the importance of validating the instrument in such contexts. Oman, with its distinctive social and cultural characteristics, provides an important context for exploring the applicability of the MHC–SF. Therefore, the present study aims to validate the Arabic version of the MHC–SF (AMHC–SF) among Omani participants by examining its factorial structure and psychometric properties.

To contextualize these measurement efforts globally, a considerable body of psychometric research has been conducted on the Mental Health Continuum across diverse countries and cultural contexts. As synthesized in the literature, these, cross-cultural validation studies of the MHC-SF have consistently demonstrated robust psychometric properties. The central empirical debate concerns its optimal factor structure, with evidence supporting both the original three-factor model (emotional, social, and psychological well-being) and a bifactor model incorporating these specific domains alongside a strong general well-being factor (e.g., the bifactor model was preferred in studies by [20,21,22,23,24,25]). The measure reliably exhibits satisfactory to excellent internal consistency across diverse populations, with total Cronbach’s alpha and McDonald’s omega coefficients typically exceeding conventional thresholds (α/ω > .70).

Furthermore, extensive validation efforts have established robust convergent validity with established well-being measures (e.g., correlations with the Satisfaction with Life Scale (SWLS) [17], and the Multidimensional Students’ Life Satisfaction Scale (MSLSS) [26] and apparent discriminant validity against instruments assessing psychological distress (e.g., negative correlations with the Hospital Anxiety and Depression Scale (HADS) [27,25] and the Depression Anxiety Stress Scales (DASS) [20], thereby affirming the scale’s capacity to capture positive mental health as a distinct construct.

A considerable body of psychometric research has been conducted on the Mental Health Continuum across diverse countries and cultural contexts. Table 1 provides a comprehensive overview of the primary validation studies, outlining sample characteristics, reliability estimates, and factor-analytic approaches. Given the diversity of populations and methodological approaches, the table serves as a concise yet detailed reference for comparing the structural and psychometric properties reported in the literature.

**Table 1:** Factor Structure and Reliability of the MHC-SF in Cross-Cultural Validations.

Study	Country & Sample	Models Tested	Best-Fitting Model	Reliability (Range & Total)
[17]	South Africa, Community (N = 1050)	1-factor, 2-factor, 3‑factor	3‑factor	α = .59-.73; α total = .74
[18]	Netherlands, Community (N = 1662)	1-factor, 2-factor, 3‑factor	3‑factor	α = .74-.83; α total = .89
[19]	Poland, Community (N = 2115)	3‑factor	3‑factor	α = .82-.87; α total = .91
[20]	India, Students (N = 539)	3‑factor	3‑factor	α = .79-.84; α total = N/R
[21]	South Africa, Students (N = 902)	1-factor, 3‑factor, bifactor	Bifactor	α = .77-.79; α total = .87
[26]	China, Students (N = 5399)	1-factor, 2-factor, 3‑factor	3‑factor	α = .83-.92; α total = .92
[22]	Serbia, Students & Adults (N = 1420)	1-factor, 2-factor, 3‑factor, bifactor	Bifactor	ω total = .81
[28]	Italy, Community (N = 1438)	1-factor, 2-factor, 3‑factor	3‑factor	α = .70-.81; α total = .86
[23]	Australia, Young Adults (N = 2220)	1-factor, 3‑factor, bifactor	Bifactor	α = .89-.91; α total = .96
[27]	Canada, Community (N = 1457)	1-factor, 2-factor, 3‑factor	3‑factor	α = .78-.90; α total = N/R
[24]	38 Countries, Students (N = 8066)	3‑factor, bifactor	Bifactor	ω = .78-.85; ω total = .91
[25]	Singapore & Australia, Community (N = 557)	1-factor, 2-factor, 3‑factor, bifactor	Bifactor	α = .89-.90; α total = .95

*Note*. α = Cronbach alpha, ω = McDonald’s omega coefficients, N/R: Not reported.

Specifically, investigating positive mental health within the Omani context provides an opportunity to evaluate how multidimensional psychometric instruments operate outside of Western, individualistic frameworks. In societies characterized by interdependent social networks and family-centric structures, an individual’s emotional and social well-being is often closely intertwined with communal harmony and familial support systems. Consequently, items on the MHC–SF that assess social integration and contribution may reflect these distinct contextual dynamics. Furthermore, given the significant role that cultural values, spiritual beliefs, and religious practices traditionally play in shaping life satisfaction and coping mechanisms in the region, responses to emotional and psychological well-being dimensions may capture unique cultural nuances. Examining these factors is essential for ensuring that the instrument is not only statistically reliable but also conceptually valid for this population.

Despite the extensive body of international research, to the best of the authors’ knowledge and based on an extensive review of major electronic databases (including PubMed, PsycINFO, Scopus, and Google Scholar) searched up to December 2025 using terms such as ‘Mental Health Continuum,’ ‘MHC-SF,’ ‘Arabic validation,’ and ‘Arab world,’ no validated Arabic version of the scale has yet been developed. The current study extends this line of inquiry by examining these psychometric properties for the Arab version (AMHC-SF) within the Omani context, thereby providing crucial evidence regarding its cross-cultural applicability and utility in the Arab world. Given the importance of culturally appropriate assessment tools, adapting and validating this scale for use in Arabic-speaking populations is essential. Previous research has emphasized that the conceptualization and experience of well-being are shaped by cultural norms and values, underscoring the need for cross-cultural validation [29–31].

The present study, therefore, aims to translate the scale into Arabic and to examine its psychometric properties in the Omani context. Specifically, the study seeks to (a) evaluate the factorial structure of the scale within the Arab cultural setting, (b) assess its reliability across the three dimensions of emotional, social, and psychological well-being, and (c) establish its discriminant validity in relation to scales of anxiety. By achieving these objectives, the study contributes to filling a critical gap in the literature. It provides researchers and practitioners in the Arab world with a reliable and valid tool for assessing positive mental health.

## Method

### Participants and data collection

This cross-sectional study was conducted from April 11, 2025, to July 8, 2025. To recruit the community-based sample in Oman, a non-probabilistic convenience sampling strategy was utilized. The survey invitation link was distributed electronically through institutional networks and popular social media platforms (e.g., WhatsApp and Twitter/X) to reach a broad audience. Additionally, a snowballing technique was encouraged, asking initial respondents to forward the link to their personal and social networks. The final sample consisted of 1101 participants, whose ages ranged from 18 to 65 years (M = 31.49, SD = 9.69). Females represented a higher proportion of the sample (59.4%) compared to males (40.6%). About one-third of the participants were students (33.2%), and half held a bachelor’s degree (50.0%). Married individuals constituted the largest marital group (50.3%).

Data were collected electronically using Google Forms, which included a digital informed consent process where participants actively confirmed their agreement by checking a mandatory box before accessing the questionnaire. Participation was entirely voluntary, and participants could withdraw at any time without penalty. Ethical approval for this study was obtained from the Research Ethics and Biosafety Committee at A’Sharqiyah University, Oman (UREBC) (Approval code: ASU/UREBC/25/141). Regarding data integrity, all items on the electronic questionnaire were set as ‘mandatory’ within Google Forms, which effectively prevented the occurrence of missing data among completed submissions. A total of 1,142 individuals accessed the survey link; however, 41 responses were incomplete or discontinued before submission (a drop-out rate of 3.59%). These incomplete responses were automatically excluded by the system, resulting in a final, fully complete dataset of 1,101 participants for subsequent psychometric analysis. Detailed demographic characteristics are presented in Table 2.

**Table 2:** Demographic Characteristics of the Study Sample (N = 1101).

Characteristics	Category	N	%
Sex	Male	447	40.6
	Female	654	59.4
Employment status	Employed	347	31.5
	Unemployed	216	19.6
	Retired	62	5.6
	Student	365	33.2
	Self-employed	111	10.1
Education	Primary	19	1.7
	Secondary	181	16.4
	Diploma	170	15.4
	Bachelor’s	551	50.0
	Postgraduate	180	16.3
Marital status	Single	398	36.1
	Married	554	50.3
	Divorced	106	9.6
	Widowed	43	3.9
Age	< 20	99	9.0
	20-29	356	32.3
	30-39	326	29.6
	40-49	231	21.0
	≥ 50	89	8.1

### Procedure

Before administering the Arabic version of the Mental Health Continuum – Short Form (AMHC-SF), a systematic process was undertaken to ensure linguistic and cultural appropriateness. The scale was first translated into Arabic by two bilingual experts, followed by back-translation into English by two independent translators to verify conceptual equivalence. The Arabic version was then reviewed by six specialists in mental health, counseling, and psychological measurement, including four associate professors and two assistant professors, to evaluate clarity, cultural relevance, and content accuracy. Subsequently, the revised version was pilot-tested on a sample of 33 participants representative of the target population to assess readability, comprehension, and overall clarity. Feedback from the pilot study led to minor contextual and linguistic refinements in two social well-being items to enhance cultural relevance. Specifically, for the Social Integration item, the examples of a ‘community’ were adapted to include the concept of the ‘tribe’ (tribal/kinship group) to better reflect the Omani social structure, while for the Social Acceptance item, the phrase ‘people are basically good’ was adjusted to explicitly emphasize human nature (inner disposition). These brief adaptations ensured that all items were consistently and efficiently understood, thereby optimizing the instrument’s conceptual equivalence prior to the formal study rollout.

### Instruments

#### Mental Health Continuum – Short Form (MHC-SF).

The Mental Health Continuum – Short Form (MHC-SF), developed by Keyes et al. [17], was designed to comprehensively assess psychological well-being, drawing on a theoretical framework that views mental health as the presence of positive functioning rather than merely the absence of psychological disorders. It consists of 14 items that capture three dimensions of well-being—emotional, social, and psychological—rated on a six-point Likert scale ranging from 5 (every day) to 0 (never). The total score ranges from 0 to 70, with higher scores reflecting greater levels of well-being.

Emotional well-being is measured through three items, with scores ranging from 0 to 15, assessing feelings of happiness, life satisfaction, and interest in life. Social well-being is represented by five items, with scores ranging from 0 to 25, reflecting individuals’ sense of social integration, community belonging, and the perception that society is meaningful and effective. Psychological well-being is assessed through six items, with scores ranging from 0 to 30, that address personal growth, autonomy, environmental mastery, quality of relationships, and a sense of purpose in life.

The original instrument has demonstrated robust psychometric properties. Item–subscale correlations ranged between 0.73 and 0.84, while reliability coefficients for the subscales ranged from 0.79 to 0.86, with an overall reliability coefficient of 0.91. Moreover, confirmatory factor analyses supported the three-factor structure, with all model fit indices meeting recommended criteria.

#### Arabic Version (AMHC–SF).

For the present study, we used the Arabic version of the Mental Health Continuum–Short Form (AMHC–SF), which was translated and culturally adapted from the original MHC–SF. The Arabic version is provided as a Supporting Information file and is shared under a Creative Commons Attribution (CC BY 4.0) license for research and educational purposes. The Arabic items and scoring follow the same structure as the original instrument, covering the three dimensions: emotional, social, and psychological well-being.

### Omani Diagnostic Battery for Anxiety Disorders (ODBAD)

The Omani Diagnostic Battery for Anxiety Disorders (ODBAD) was developed by Al-Manji [32] for use in the Omani context, based on the Diagnostic and Statistical Manual of Mental Disorders (DSM-5-TR). Its psychometric properties were examined across both community and clinical samples, and it comprises six scales designed to assess different anxiety disorders. For the present study, three scales were employed to examine the discriminant validity of the AMHC-SF.

**The Social Anxiety Scale (SAS)** consists of 10 items rated on a five-point Likert scale ranging from 1 (never) to 5 (always), with possible scores between 10 and 50. The scale demonstrated strong reliability, with Cronbach’s alpha of .90, split-half reliability of .88, and composite reliability of .92. Confirmatory factor analysis (CFA) also indicated acceptable model fit (χ²/df = 1.87, RMSEA = .06, TLI = .90, CFI = .92).

**The Panic Disorder Scale (PDS)** is composed of 11 items, also rated on a five-point Likert scale, with scores ranging from 11 to 55. This scale showed excellent internal consistency (Cronbach’s alpha = .96), split-half reliability of .94, and composite reliability of .96. CFA indices demonstrated a good model fit (χ²/df = 2.11, RMSEA = .04, TLI = .94, CFI = .95).

**The Generalized Anxiety Scale (GAS)** includes 11 items rated on the same five-point Likert format, with a total score ranging from 11 to 55. Reliability analysis yielded a Cronbach’s alpha of .94, split-half reliability of .91, and composite reliability of .94. CFA supported the factor structure with acceptable fit indices (χ²/df = 1.98, RMSEA = .05, TLI = .90, CFI = .91).

### Data analysis

IBM SPSS Statistics (version 28) was used to conduct descriptive analyses and examine the reliability and correlations of the study measures. Means and standard deviations were calculated for all scale items. Pearson correlations, corrected for attenuation, were computed between items, subscales, and total scores. Reliability was assessed using Cronbach’s alpha and McDonald’s omega, providing estimates of internal consistency for both the total scales and subscales. Additionally, Pearson correlations between the MHC-SF total and subscale scores and the three anxiety scales (SAS, PDS, and GAS) were calculated to examine discriminant validity.

Mplus (version 8.3) was employed to perform confirmatory factor analysis (CFA) of the AMHC-SF. Four competing models were tested: single-factor, two-factor, three-factor, and bifactor structures. Model fit was evaluated using multiple indices based on established psychometric benchmarks [33,34]. Specifically, model fit was considered acceptable to good if the Chi-square divided by degrees of freedom (χ²/df) ratio was ≤ 3.0 or ≤ 5.0, and if the Comparative Fit Index (CFI) and the Tucker-Lewis Index (TLI) values were ≥ 0.90 (ideally ≥ 0.95). Additionally, a Root Mean Square Error of Approximation (RMSEA) RMSEA ≤ 0.06 to 0.08 (alongside its 90% confidence interval) and a Standardized Root Mean Square Residual (SRMR) ≤ 0.08 were required to confirm an adequate model fit. For model comparison (e.g., comparing the three-factor model with the bifactor structure), lower values of the Akaike Information Criterion (AIC) and the Bayesian Information Criterion (BIC) indicated a more parsimonious and superior fit, alongside evaluation of standardized factor loadings. The bifactor model allowed examination of a general factor alongside subscale factors, providing insight into the measure’s multidimensionality and enabling a more comprehensive assessment of model fit than single- or correlated-factor solutions.

Prior to conducting the Confirmatory Factor Analysis (CFA), the data were evaluated considering the scale characteristics of the MHC–SF items (6-point Likert scale). To account for potential deviations from multivariate normality common in ordinal behavioral data, the Confirmatory Factor Analysis was executed using the Robust Maximum Likelihood (MLR) estimator. The MLR estimator provides standard errors and chi-square test statistics (the Yuan-Bentler scaled (χ²) that are robust to non-normality, thereby preventing the inflation of values and ensuring the distortion-free estimation of model fit indices [35].

R was used to calculate bifactor-specific indices for the AMHC-SF, including Explained Common Variance (ECV), Omega Hierarchical (ωH), and H (construct replicability) using the *BifactorIndicesCalculator* package. Omega hierarchical (ωH) was computed for the general factor to estimate the proportion of variance in total scores attributable to it after controlling for subscale factors, indicating the extent to which the total score reflects a single general dimension. In contrast, H was calculated for both the general factor and the subscale factors to assess the strength and replicability of each factor, showing how well each factor is represented by its items and its stability across different samples. These indices were used to evaluate the relative contribution of the general factor versus subscale factors and to interpret the dimensionality of the scale.

## Results

### Descriptive Statistics, item analysis, and reliability

Table 3 presents the descriptive statistics and corrected item-total correlations for all items of the Arabic Mental Health Continuum – Short Form (AMHC-SF), along with reliability indices for the subscales and the total scale. The table provides information on the mean scores and standard deviations of each item, the strength of each item’s contribution to its respective subscale, and the overall internal consistency of the instrument. Additionally, correlations between the three subscales—emotional, social, and psychological well-being—are reported, highlighting the interrelationships among the dimensions of the scale.

**Table 3:** AMHC-SF Item Means, Correlations, and Reliability.

Item	Mean	SD	Skew	Kurt	Emotional	Social	Psychological	Well-Being
					Corrected Item-Total Correlation			
E1	3.42	1.26	−0.72	0.20	0.71			0.69
E2	3.61	1.36	−0.89	0.49	0.75			0.73
E3	3.66	1.48	−0.95	0.35	0.74			0.74
S1	3.24	1.59	−0.64	−0.77		0.59		0.67
S2	3.72	1.56	−0.81	0.02		0.70		0.74
S3	3.35	1.62	−0.77	−0.60		0.78		0.73
S4	3.19	1.60	−0.61	−0.81		0.71		0.68
S5	3.17	1.58	−0.66	−0.70		0.74		0.71
P1	3.65	1.41	−0.75	0.25			0.77	0.77
P2	3.65	1.42	−0.91	0.11			0.80	0.77
P3	3.54	1.52	−0.97	−0.16			0.76	0.76
P4	3.62	1.50	−0.98	−0.13			0.75	0.69
P5	3.63	1.46	−0.83	0.07			0.80	0.75
P6	3.76	1.45	−0.81	0.54			0.80	0.78
Internal consistency								
Cronbach’s alpha (α)					0.86	0.88	0.92	0.95
McDonald’s Omega (ω)					0.86	0.88	0.92	0.95
Correlation between dimensions and total score								
Emotional					1.00	0.74^**^	0.72^**^	0.86^**^
Social						1.00	0.75^**^	0.91^**^
Psychological							1.00	0.92^**^

As shown in Table 3, the items of the AMHC-SF demonstrated adequate means, with most items scoring above the midpoint of the scale, indicating relatively high levels of well-being among the participants. Univariate distribution analyses indicated that skewness indices ranged from −0.98 to −0.61, and kurtosis indices ranged from −0.81 to 0.54. These values fall strictly within the acceptable psychometric thresholds for normal-theory frameworks (i.e., absolute skewness < 2.0 and kurtosis < 7.0), representing a mild to moderate negative skew that is well-handled by the robust estimation procedures.

Corrected item-total correlations ranged from 0.59 to 0.80 for the subscales, suggesting that each item was positively and meaningfully associated with its respective dimension. Furthermore, correlations between each item and the total scale scores were similarly strong (ranging from 0.67 to 0.78), demonstrating that all items contributed appropriately to the overall measure. Reliability coefficients were high, with Cronbach’s alpha values ranging from 0.86 to 0.92 for the subscales and 0.95 for the total scale. Similarly, McDonald’s omega values confirmed strong internal consistency, supporting the reliability of both the subscales and the overall measure. Correlations between the subscales were also substantial, ranging from 0.72 to 0.75, while correlations between individual dimensions and the total score ranged from 0.86 to 0.92, indicating strong associations among the emotional, social, and psychological well-being dimensions.

### Structural validity

**Fig 1:**
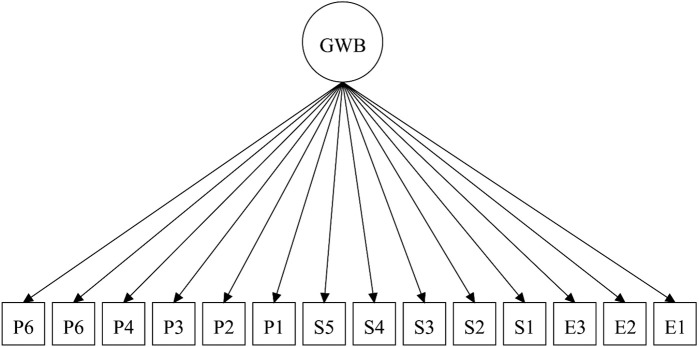
Single-factor model of the MHC-SF. *Note*. GWB = general well-being.

**Fig 2:**
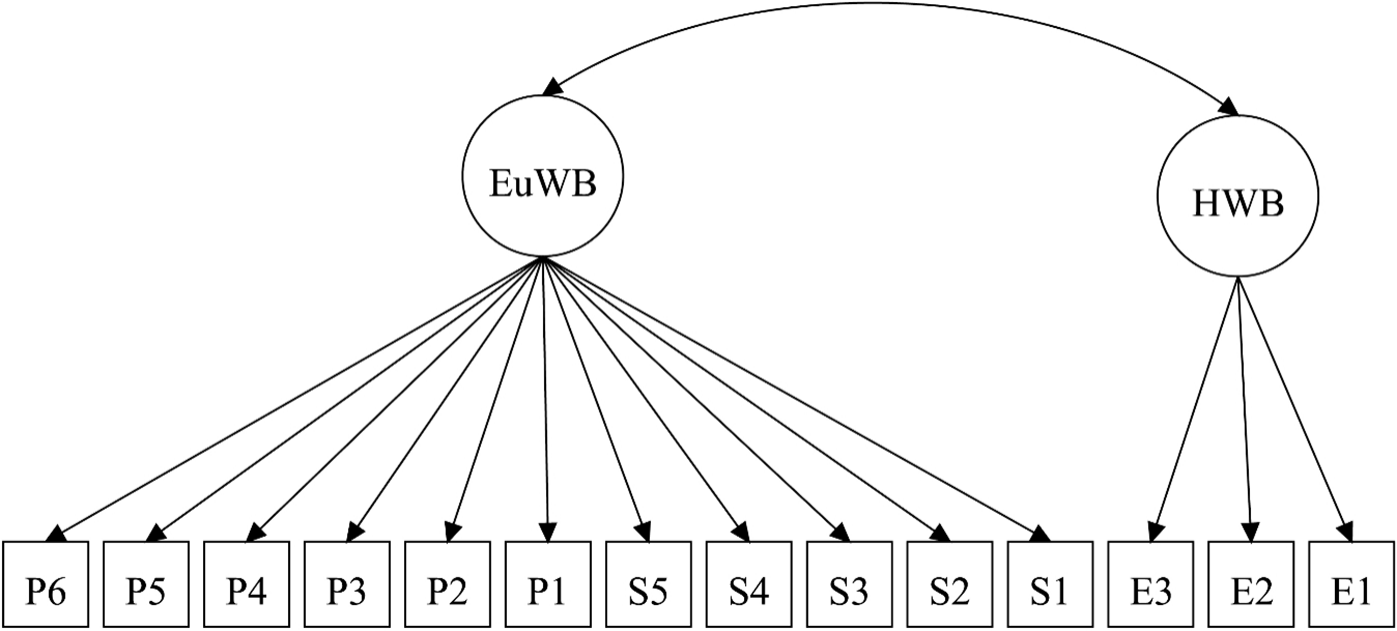
Two-factor model of the MHC-SF. *Note*. HWB = hedonic well-being, EuWB = eudaimonic well-being.

**Fig 3:**
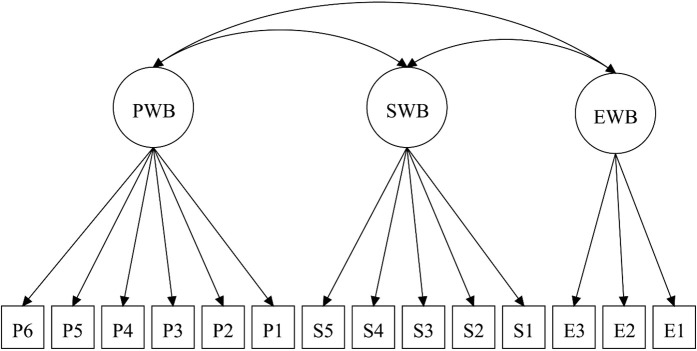
Three-factor model of the MHC-SF. *Note*. EWB = emotional well-being, SWB = social well-being, PWB = psychological well-being.

**Fig 4:**
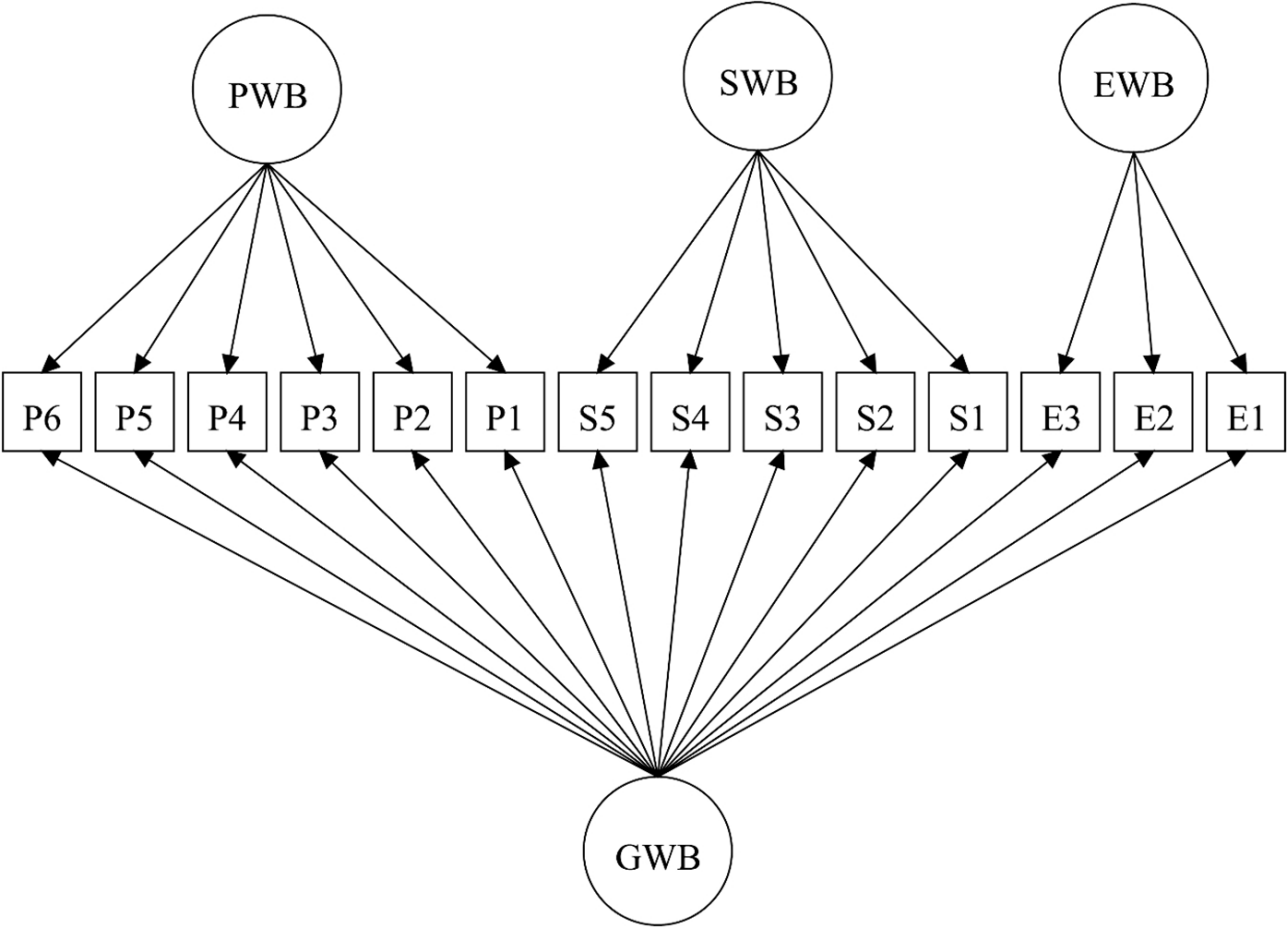
Bifactor model of the MHC-SF. *Note*. EWB = emotional well-being, SWB = social well-being, PWB = psychological well-being, GWB = general well-being.

Based on theoretical considerations and prior research on the Mental Health Continuum – Short Form (e.g., [21,22,23,24,25]), the current study examined four competing CFA models of the Arabic version of the scale (AMHC-SF) to determine the most appropriate representation of psychological well-being in an Omani community sample. The first model was a single-factor model, assuming that all 14 items reflect a single general well-being construct (Fig 1). The second model was a two-factor model, with emotional well-being (items 1–3) and combined social and psychological well-being (items 4–14), which reflected a simplified distinction between hedonic and eudaimonic aspects (Fig 2). The third model was a three correlated-factors model, separating emotional (EWB, items 1–3), social (SWB, items 4–8), and psychological well-being (PWB, items 9–14), consistent with the theoretical dimensions of the original scale (Fig 3). Finally, the bifactor model included a general well-being factor (GWB) and three orthogonal group factors, capturing the unique variance of EWB, SWB, and PWB, which allowed for the simultaneous evaluation of overall and specific well-being dimensions (Fig 4).

Table 4 presents the fit indices for these four models, including χ², χ²/df, RMSEA, SRMR, CFI, and TLI, which were used to evaluate how well each model represents the underlying factor structure of the AMHC-SF. Fit indices were interpreted according to commonly recommended thresholds: χ²/df ≤ 5 indicates acceptable fit and < 2 excellent fit; RMSEA ≤ 0.08 indicates acceptable and ≤ 0.05 excellent; SRMR ≤ 0.08 indicates acceptable and ≤ 0.05 excellent; CFI and TLI ≥ 0.90 indicate acceptable and ≥ 0.95 excellent [33,34]. This presentation allows for identification of the model that best captures both the general well-being construct and its specific subdimensions, providing a foundation for subsequent analyses.

**Table 4:** Fit Indices for Competing Confirmatory Factor Analysis Models of the AMHC-SF.

Model	χ^2^ (df)	χ^2^/df	RMSEA	SRMR	CFI	TLI	AIC	BIC
Single factor	771.32 (77)	10.02	0.090	0.052	0.88	0.86	46294.06	46504.23
Two factors	601.91 (76)	7.92	0.079	0.048	0.91	0.89	45984.26	46199.43
Three factors	283.82 (74)	3.84	0.051	0.035	0.96	0.96	45412.61	45637.79
Bifactor	124.33 (63)	1.97	0.032	0.017	0.99	0.98	45168.29	45448.51

*Note*. χ^2^ = chi square, df = degree of freedom, RMSEA = Root Mean Square Error of Approximation, SRMR = Standardized Root Mean Square Residual, CFI = Comparative Fit Index, TLI = Tucker-Lewis Index, AIC = Akaike Information Criterion, BIC = Bayesian Information Criterion.

As detailed in Table 4, the single-factor model showed poor fit, indicating that a unidimensional structure does not adequately capture the multidimensional nature of psychological well-being. The two correlated-factors model improved fit but still fell short of thresholds for excellent fit, suggesting partial representation of the underlying structure. Conversely, the three correlated-factors model, representing emotional, social, and psychological well-being, provided a substantially better fit (CFI = 0.96, TLI = 0.96, RMSEA = 0.051), supporting the theoretical distinction among these subdimensions. Finally, the bifactor model demonstrated the best overall descriptive fit across all indices (CFI = 0.99, TLI = 0.98, RMSEA = 0.032), while also exhibiting the lowest AIC and BIC values. To formally assess the conceptual plausibility and statistical superiority of these competing configurations, nested model comparisons were conducted. Given the utilization of the MLR estimator, formal testing was executed via the Satorra–Bentler scaled chi-square difference test (Δχ^2^), supplemented by observing changes in the Comparative Fit Index (ΔCFI) and relative improvements in information criteria (ΔAIC and ΔBIC). In alignment with established psychometric benchmarks [36], CFI > 0.01 was utilized alongside a statistically significant Δχ^2^ as empirical evidence of meaningful incremental fit improvement. Additionally, a decrease in AIC and BIC values exceeding 10 was adopted as decisive evidence of superior model parsimony [37]. The formal statistical differentials for these comparisons are presented in Table 5.

**Table 5:** Model Comparison and Fit Differentials for the Competing Structures.

Models	Δχ^2^	Δdf	p	ΔCFI	ΔAIC	ΔBIC
1-factor vs 2-factors	90.71	1	< 0.001	0.03	−309.80	−304.80
2-factor vs 3-factor	185.34	2	< 0.001	0.05	−571.65	−561.64
3-factor vs Bifactor	144.97	11	< 0.001	0.03	−244.32	−189.28

The formal comparisons in Table 5 confirm that the transition from the baseline single-factor model to the alternative two-factor model demonstrated an initial improvement (Δχ^2^ (1) = 90.71, p < 0.001, ΔCFI = 0.03, ΔAIC = −309.80; ΔBIC = −304.80), which was further enhanced when shifting to the theoretical target three-factor model, yielding a highly substantial and statistically significant structural progression, (Δχ^2^ (2) = 185.34, p < 0.001, ΔCFI = 0.03). This shift was concurrently supported by a massive drop in descriptive information criteria (ΔAIC = −571.65, ΔBIC = −561.64).

More importantly, to address the core incremental validity of the general well-being dimension, the bifactor model was formally tested against the target three-factor framework. The formal Satorra–Bentler difference test confirmed that the introduction of the general factor resulted in a statistically significant incremental improvement in fit, Δχ^2^(11) = 144.97, p < 0.001. This significant chi-square reduction operated in tandem with a notable shift in descriptive statistics (ΔCFI = 0.03) and a substantial decrease in information criteria (ΔAIC = −244.32, ΔBIC = −189.28).

In conclusion, although the three-factor model reasonably represents the subdimensions, the formal statistical and information-theoretic diagnostics provide robust empirical support for the structural superiority of the bifactor framework, confirming it offers the most comprehensive, robust, and parsimonious structure for the AMHC-SF within the sampled population.

**Fig 5:**
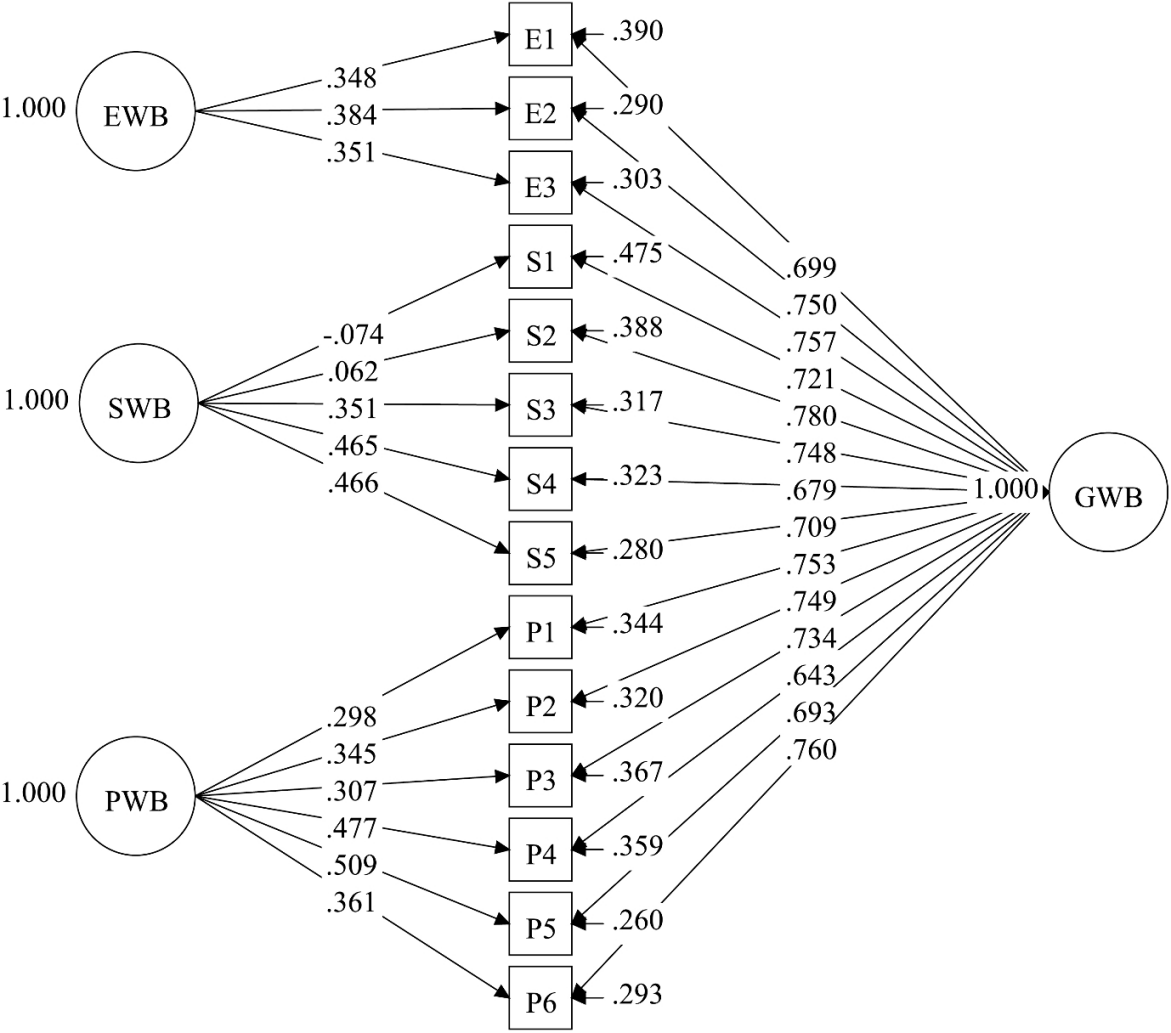
Bifactor loadings of AMHC-SF. *Note*. EWB = emotional well-being, SWB = social well-being, PWB = psychological well-being, GWB = general well-being.

Table 6 and Fig 5 present the factor loadings and psychometric indices of the Arabic Mental Health Continuum – Short Form (AMHC-SF) based on the bifactor model. The first section of the table shows the standardized loadings of each item on the general well-being factor (GWB) and the three specific subfactors: emotional well-being (EWB), social well-being (SWB), and psychological well-being (PWB). The second section reports the bifactor indices, including MacDonald’s omega hierarchical (ωH) for the general factor, omega subscale (ωS) for the specific factors, explained common variance (ECV) in three forms—ECV_SS (variance explained by each specific factor from its own items), ECV_SG (variance explained by each specific factor relative to the general factor), and ECV_GS (variance explained by the general factor for each specific factor)—and construct reliability (H) for both the general and specific factors. These indices collectively provide information on the proportion of variance attributable to the general and specific factors, the reliability of the scale and its subdimensions, and the extent to which the AMHC-SF captures both a general well-being construct and its multidimensional components.

**Table 6:** Standardized factor loadings and bifactor indices for the AMHC-SF.

Item / Indicator	GWB	EWB	SWB	PWB
E1. happy	0.70	0.35		
E2. interested in life	0.75	0.38		
E3. satisfied with life	0.76	0.35		
S1. that you had something important to contribute to society	0.72		−0.07	
S2. that you belonged to a community (like a social group, your neighborhood, your city)	0.78		0.06	
S3. that our society is becoming a better place for all people	0.75		0.35	
S4. that people are basically good	0.68		0.47	
S5. that the way our society works makes sense to you	0.71		0.47	
P1. that you liked most parts of your personality	0.75			0.30
P2. good at managing the responsibilities of your daily life	0.75			0.35
P3. that you had warm and trusting relationships with others	0.73			0.31
P4. that you had experiences that challenged you to grow and become a better person	0.64			0.48
P5. confident to think or express your own ideas and opinions	0.69			0.51
P6. that your life has a sense of direction or meaning to it	0.76			0.36
MacDonald’s ωH	0.89			
MacDonald’s ωS		0.17	0.10	0.20
ECV_SS	0.80	0.19	0.18	0.23
ECV_GS	0.80	0.81	0.82	0.77
H	0.94	0.31	0.41	0.53

*Note*. ωH = omega hierarchical, ωS = omega subscale, ECV_SS = explained common variance by specific factors, ECV_GS = explained common variance by general factors, H = construct reliability. EWB = emotional well-being, SWB = social well-being, PWB = psychological well-being, GWB = general well-being

As shown in Table 6, all items exhibited substantial loadings on the general well-being factor (GWB), ranging from 0.64 to 0.76, which indicates that the AMHC-SF predominantly reflects a unidimensional construct of general well-being. The loadings on the specific subfactors—emotional well-being (EWB), social well-being (SWB), and psychological well-being (PWB)—were generally smaller, with some items showing low or near-zero contributions. This pattern suggests that while the subdimensions capture unique variance beyond the general factor, the majority of the shared variance among items is accounted for by the general factor.

The bifactor indices provide additional support for this interpretation. MacDonald’s omega hierarchical (ωH) for the general factor was high (0.89), indicating strong reliability and suggesting that the AMHC-SF can be meaningfully interpreted at the total scale level. In contrast, omega subscale (ωS) values for the specific factors were lower (0.10–0.20), reflecting limited variance uniquely attributable to each subdimension after accounting for the general factor. This is further supported by the explained common variance (ECV) values: the general factor explains the majority of common variance across items (ECV_GS = 0.77–0.82), whereas the specific factors contribute smaller proportions (ECV_SS = 0.18–0.23). These findings indicate that, although the subfactors provide supplementary information, the general well-being dimension is the dominant source of shared variance.

Finally, construct reliability (H) was very high for the general factor (0.94) and moderate for the subfactors (0.31–0.53), confirming that the AMHC-SF is a highly reliable instrument for measuring overall well-being while retaining some capacity to capture the emotional, social, and psychological subcomponents. Overall, these results support the interpretation of the AMHC-SF primarily at the general factor level, with the subfactors providing meaningful but secondary information for more nuanced assessments of well-being.

Regarding the standardized factor loadings of the bifactor model, a specific psychometric pattern was observed for the Social Well-Being (SWB) specific factor. Notably, items S1 (λ = −0.07) and S2 (λ = 0.06) exhibited factor loadings that approached zero on their designated specific dimension. From a psychometric standpoint within a bifactor framework, near-zero loadings on a specific factor indicate that the variance of these particular items is almost entirely absorbed by the overarching General Well-Being factor, leaving negligible residual variance unique to the subdimension itself [38,39]. Rather than indicating a structural defect or a requirement for item elimination, retaining these items was psychometrically justified to preserve the theoretical breadth and content validity of the comprehensive instrument, as they continue to contribute robustly to the global construct.

### Discriminant validity

Table 7 shows the correlations between the Arabic Mental Health Continuum – Short Form (AMHC-SF) subscales and total scores and three anxiety scales (Social Anxiety Scale, Panic Disorder Scale, and Generalized Anxiety Scale). The table was used to examine the discriminant validity of the AMHC-SF, specifically to determine whether the scale measures a construct distinct from anxiety-related symptoms.

**Table 7:** Correlations Between AMHC-SF Subscales and Total Score with Anxiety Measures.

	Emotional	Social	Psychological	Well-Being
Social Anxiety	−0.46^**^	−0.45^**^	−0.55^**^	−0.54^**^
Panic Disorder	−0.45^**^	−0.41^**^	−0.45^**^	−0.48^**^
Generalized Anxiety	−0.54^**^	−0.53^**^	−0.55^**^	−0.59^**^

** *p-*value < 0.05

As shown in Table 7, all correlations between the AMHCSF subscales and total score with the three anxiety measures were negative and statistically significant (p < 0.01), ranging from −0.41 to −0.59. These negative correlations indicate that higher levels of psychological well-being are associated with lower levels of social anxiety, panic, and generalized anxiety, supporting the discriminant validity of the AMHC-SF. The magnitude of these correlations suggests that while the constructs are related, they measure distinct psychological domains, confirming that the AMHC-SF captures positive aspects of mental health that are conceptually different from anxiety symptoms.

## Discussion

The current study aimed to validate the Arabic version of the Mental Health Continuum–Short Form (AMHC-SF) in an Omani community sample, focusing on its factorial structure, reliability, and discriminant validity. Overall, the findings provide strong evidence for the psychometric soundness of the scale in Arabic-speaking populations, while also highlighting some unique cultural and contextual considerations that differentiate the present results from prior international studies.

Confirmatory factor analyses revealed that the bifactor model offered the best fit for the AMHC-SF, capturing a general well-being factor (GWB) alongside three theoretically grounded subdimensions: emotional well-being (EWB), social well-being (SWB), and psychological well-being (PWB). Standardized loadings on the general factor were substantial (0.64–0.76), indicating that the AMHC-SF primarily reflects a robust general construct of positive mental health. However, loadings on the specific subfactors were relatively lower than those reported in several prior studies, with some items showing near-zero contributions, particularly within the SWB dimension [21,22,23,24,25]. This weaker differentiation among the subdimensions suggests that, in the Omani context, emotional, social, and psychological aspects of well-being may be more interconnected or less distinctly perceived by respondents than in Western or other non-Western populations.

This specific psychometric pattern can be conceptually framed through the lens of cross-cultural well-being research. In collectivistic cultural frameworks, such as that of the Sultanate of Oman, the boundaries between individual social functioning and global life satisfaction are often highly permeable. Cross-cultural literature consistently suggests that in interdependent contexts, social connectedness, community integration, and social contribution are not perceived as peripheral or isolated domains of wellness; rather, they are foundational, constitutive elements of general psychological flourishing [30,40].

However, because the current study did not empirically measure collectivistic tendencies or cultural values at the individual level, this socio-cultural interpretation should be treated as a plausible post-hoc hypothesis rather than a definitive empirical conclusion. It is highly possible that for Omani participants, functioning well socially is structurally synonymous with flourishing generally, which would explain why the variance of these specific items was entirely absorbed by the global well-being factor. Future cross-cultural validation studies should formally integrate explicit measures of cultural orientation (e.g., idiom of interdependence vs. independence) alongside the AMHC-SF to empirically test whether cultural collectivism systematically accounts for the attenuation of specific factor loadings in non-Western contexts.

Several factors may account for this pattern. First, cultural norms and values in Oman may promote a holistic perception of well-being, integrating emotional, social, and psychological dimensions into a unified construct. This is consistent with research showing that collectivist cultures often perceive well-being as a blend of personal and social harmony rather than as discrete domains [29–31]. Second, the process of linguistic translation and adaptation of the MHC-SF may have influenced item interpretation. Despite careful translation and back-translation procedures, subtle semantic differences may reduce the ability of specific items to capture distinct facets of well-being, a challenge observed in cross-cultural psychometrics [18,26]. Third, the use of a community sample with relatively homogeneous well-being levels might have limited variability, contributing to lower factor-specific loadings. Homogeneity effects have been reported in other validation studies in non-Western contexts [20,41].

Despite these limitations, the general well-being factor remained robust, consistent with Keyes’ [1] conceptualization of mental health as a multidimensional yet integrated construct. The predominance of the general factor aligns with findings from Australia [23], South Africa [21], and multi-country studies [24], which indicate that total MHC-SF scores can meaningfully represent overall positive mental health, even when subdimension differentiation is weaker.

Internal consistency was high, with Cronbach’s alpha ranging from 0.86 to 0.92 for subscales and 0.95 for the total scale, and McDonald’s omega coefficients showing similar strength (0.86–0.95). These results are in line with prior studies, including [18,26,25], confirming that the AMHC-SF is a reliable instrument for both total and subscale scores. The substantial correlations among subscales (r = 0.72–0.75) and the total score (r = 0.86–0.92) further suggest that, while the subdimensions are theoretically distinct, they are empirically interrelated, reflecting an integrated view of well-being consistent with cross-cultural research [17,41].

The correlations between the AMHC-SF and three anxiety scales—social anxiety, panic disorder, and generalized anxiety—were all negative and statistically significant (r = −0.41 to −0.59, p < 0.01), supporting the discriminant validity of the instrument. These findings indicate that higher psychological well-being is associated with lower anxiety, reflecting that positive mental health and psychopathology, while related, are conceptually distinct [1,10]. According to DSM-5-TR, anxiety disorders involve excessive worry and heightened physiological responses, causing distress and impairing psychological and social functioning [42]. In contrast, psychological well-being represents a state of happiness, life satisfaction, purposeful engagement, and ongoing personal growth [43,44]. Thus, well-being can be considered a positive state opposite to anxiety, characterized by balance and adaptive functioning, while anxiety entails excessive fears and negative emotions. Supporting this conceptualization, previous studies reported similar inverse associations between anxiety and well-being, including [45–47], who found comparable results for social anxiety in university students. Moreover, interventions enhancing well-being have been shown to reduce anxiety and improve emotional regulation and adaptive functioning [48–50], highlighting the practical implications of promoting positive mental health as a buffer against anxiety.

Bifactor indices offer additional insight into the scale’s structure. The general factor’s omega hierarchical (ωH = 0.89) indicates that the AMHC-SF can be interpreted with high confidence at the total scale level. Omega subscale values for the specific dimensions were lower (0.10–0.20), reflecting limited variance uniquely attributable to subfactors, while explained common variance (ECV) analyses showed that the general factor accounted for 77–82% of item variance. Construct reliability for the general factor was high (H = 0.94), whereas subfactor reliabilities ranged from moderate to low (0.31–0.53). This pattern suggests that while subdimensions provide additional nuanced information, the scale is most robustly interpreted as an overall measure of positive mental health. These results mirror findings from multi-country studies [24,25], highlighting the general factor’s predominance in capturing global well-being across cultural contexts.

The study provides several key implications. Theoretically, the findings support Keyes’ [1] multidimensional model of mental health while emphasizing the dominance of general well-being in certain cultural contexts. Practically, the AMHC-SF offers a brief, reliable, and culturally adapted tool for assessing positive mental health in Arabic-speaking populations. It is suitable for both research and applied settings, including community surveys, clinical assessments, and intervention monitoring. The negative associations with anxiety highlight the scale’s potential utility in identifying individuals at risk and informing well-being–oriented preventive and therapeutic programs.

Overall, the study confirms that the AMHC-SF captures both the general essence of well-being and the distinct contributions of emotional, social, and psychological domains, even when the subfactors exhibit weaker differentiation. These findings advance cross-cultural understanding of well-being measurement, emphasizing the need to consider cultural and contextual influences on the expression and structure of positive mental health.

### Limitations and recommendations

Despite these encouraging findings, several limitations should be noted. The study employed a cross-sectional design, which limits causal interpretations and structural stability assessments over time due to the lack of test-retest reliability data. Additionally, all measures relied on self-report instruments, which are inherently vulnerable to social desirability and recall biases. Methodologically, discriminant validity was evaluated using the Omani Digital Behavioral Anxiety Detector (ODBAD)—an as-yet unpublished anxiety battery—which may constrain immediate comparability. Furthermore, the online recruitment format introduces potential selection biases, potentially underrepresenting older adults, individuals with lower digital literacy, and rural Omani populations. Finally, although a collectivistic cultural framework was hypothesized to explain the near-zero specific factor loadings in the bifactor model, individual cultural orientation was not empirically measured.

Future research should utilize longitudinal and test-retest designs to establish the temporal stability and predictive validity of the A–MHC–SF. To enhance generalizability, future studies should employ diversified, field-based sampling strategies (e.g., paper-and-pencil administrations) to ensure the inclusion of older demographics and remote, rural communities across Oman. Moreover, integrating multi-informant measures alongside fully published, validated clinical criteria would provide a more comprehensive validation framework. Crucially, future psychometric evaluations should formally integrate explicit scales measuring cultural orientation (individualism-collectivism) to empirically test the cultural blending of well-being dimensions, while exploring advanced bifactor variance partition indices to ensure subfactor stability.

## Conclusion

In summary, the Arabic version of the Mental Health Continuum–Short Form (AMHC-SF) demonstrates robust psychometric properties, including strong reliability, clear factorial structure, and satisfactory discriminant validity. The bifactor model highlights the predominance of general well-being while retaining meaningful subdimensions, supporting both total and domain-specific score interpretations. These results provide researchers and practitioners in Oman and the broader Arab world with a reliable and culturally sensitive instrument for assessing positive mental health, facilitating both research and applied interventions aimed at promoting flourishing and resilience across the lifespan.

## Supporting information

S1 File English version scale. (PDF)

## References

[1] Keyes CLM. The mental health continuum: from languishing to flourishing in life. J Health Soc Behav. 2002;43(2):207–22. doi: 10.2307/3090197 12096700

[2] GBD 2017 Disease and Injury Incidence and Prevalence Collaborators. Global, regional, and national incidence, prevalence, and years lived with disability for 354 diseases and injuries for 195 countries and territories, 1990–2017: A systematic analysis for the Global Burden of Disease Study 2017. The Lancet. 2018;392(10159):1789–858. doi: 10.1016/S0140-6736(18)32279-7 PMC6227754 30496104

[3] Fuehrer A. The neglect of mental health research in the global health agenda. Global Mental Health Journal. 2019;6:e15. doi: 10.1017/gmh.2019.15

[4] Story CM. The growing crisis of youth mental health: Causes and consequences. Journal of Youth Studies. 2010;13(4):419–35. doi: 10.1080/13676261003801793

[5] Kessler RC, Berglund P, Demler O, Jin R, Merikangas KR, Walters EE. Lifetime prevalence and age-of-onset distributions of DSM-IV disorders in the National Comorbidity Survey Replication. Arch Gen Psychiatry. 2005;62(6):593–602. doi: 10.1001/archpsyc.62.6.593 15939837

[6] Keeling DI. Children at risk: Attachment and the long-term effects on mental health. Child and Adolescent Mental Health. 2011;16(4):202–9. doi: 10.1111/j.1475-3588.2011.00621.x

[7] Kitzerew S. Academic stress in adolescents: Sources and consequences. Journal of Adolescent Research. 2003;18(2):178–90. doi: 10.1177/0743558402250345

[8] Arnett JJ. Emerging adulthood: The winding road from the late teens through the twenties. 2nd ed. Oxford University Press.

[9] Cornwell EY, Waite LJ. Social disconnectedness, perceived isolation, and health among older adults. J Health Soc Behav. 2009;50(1):31–48. doi: 10.1177/002214650905000103 19413133 PMC2756979

[10] Ryff CD. Psychological well-being revisited: advances in the science and practice of eudaimonia. Psychother Psychosom. 2014;83(1):10–28. doi: 10.1159/000353263 24281296 PMC4241300

[11] Rogers CH, Floyd FJ, Seltzer MM, Greenberg J, Hong J. Long-term effects of the death of a child on parents’ adjustment in midlife. J Fam Psychol. 2008;22(2):203–11. doi: 10.1037/0893-3200.22.2.203 18410207 PMC2841012

[12] Greenfield EA, Marks NF. Identifying experiences of physical and psychological violence in childhood that jeopardize mental health in adulthood. Child Abuse Negl. 2010;34(3):161–71. doi: 10.1016/j.chiabu.2009.08.012 20223518 PMC2838932

[13] Marks NF. Does It Hurt to Care? Caregiving, Work-Family Conflict, and Midlife Well-Being. Journal of Marriage and the Family. 1998;60(4):951. doi: 10.2307/353637

[14] Jieun S, Nadine FM, Gyounghae H. Work, Family, Work-Family Spillover and Mental Health among Working Adults -A Comparison of Data from National Surveys in Korea and the U.S.-. Family and Culture. 2007;19(2):61–92. doi: 10.21478/FAMILY.19.2.200706.003

[15] Son J, Wilson J. Volunteer Work and Hedonic, Eudemonic, and Social Well‐Being. Sociological Forum. 2012;27(3):658–81. doi: 10.1111/j.1573-7861.2012.01340.x

[16] Greenfield EA, Marks NF. Formal volunteering as a protective factor for older adults’ psychological well-being. J Gerontol B Psychol Sci Soc Sci. 2004;59(5):S258-64. doi: 10.1093/geronb/59.5.s258 15358800

[17] Keyes CLM, Wissing M, Potgieter JP, Temane M, Kruger A, van Rooy S. Evaluation of the mental health continuum-short form (MHC-SF) in setswana-speaking South Africans. Clin Psychol Psychother. 2008;15(3):181–92. doi: 10.1002/cpp.572 19115439

[18] Lamers SMA, Westerhof GJ, Bohlmeijer ET, ten Klooster PM, Keyes CLM. Evaluating the psychometric properties of the Mental Health Continuum-Short Form (MHC-SF). J Clin Psychol. 2011;67(1):99–110. doi: 10.1002/jclp.20741 20973032

[19] Karaś D, Cieciuch J, Keyes CLM. The Polish adaptation of the Mental Health Continuum-Short Form (MHC-SF). Personality and Individual Differences. 2014;69:104–9. doi: 10.1016/j.paid.2014.05.011

[20] Singh K, Bassi M, Junnarkar M, Negri L. Mental health and psychosocial functioning in adolescence: an investigation among Indian students from Delhi. J Adolesc. 2015;39:59–69. doi: 10.1016/j.adolescence.2014.12.008 25588610

[21] de Bruin GP, du Plessis GA. Bifactor analysis of the mental health continuum-short form (MHC-SF). Psychol Rep. 2015;116(2):438–46. doi: 10.2466/03.02.PR0.116k20w6 25730745

[22] Jovanović V. Structural validity of the Mental Health Continuum-Short Form: The bifactor model of emotional, social and psychological well-being. Personality and Individual Differences. 2015;75:154–9. doi: 10.1016/j.paid.2014.11.026

[23] Hides L, Quinn C, Stoyanov S, Cockshaw W, Mitchell T, Kavanagh DJ. Is the mental wellbeing of young Australians best represented by a single, multidimensional or bifactor model?. Psychiatry Res. 2016;241:1–7. doi: 10.1016/j.psychres.2016.04.077 27152903

[24] Żemojtel-Piotrowska M, Piotrowski JP, Osin EN, Cieciuch J, Adams BG, Ardi R, et al. The mental health continuum-short form: The structure and application for cross-cultural studies-A 38 nation study. J Clin Psychol. 2018;74(6):1034–52. doi: 10.1002/jclp.22570 29380877

[25] Yeo ZZ, Suárez L. Validation of the mental health continuum-short form: The bifactor model of emotional, social, and psychological well-being. PLoS One. 2022;17(5):e0268232. doi: 10.1371/journal.pone.0268232 35584145 PMC9116639

[26] Guo C, Tomson G, Guo J, Li X, Keller C, Söderqvist F. Psychometric evaluation of the Mental Health Continuum-Short Form (MHC-SF) in Chinese adolescents - a methodological study. Health Qual Life Outcomes. 2015;13:198. doi: 10.1186/s12955-015-0394-2 26651829 PMC4676120

[27] Doré I, O’Loughlin JL, Sabiston CM, Fournier L. Psychometric Evaluation of the Mental Health Continuum-Short Form in French Canadian Young Adults. Can J Psychiatry. 2017;62(4):286–94. doi: 10.1177/0706743716675855 28363262 PMC5407549

[28] Petrillo G, Capone V, Caso D, Keyes CLM. The Mental Health Continuum–Short Form (MHC–SF) as a Measure of Well-Being in the Italian Context. Soc Indic Res. 2014;121(1):291–312. doi: 10.1007/s11205-014-0629-3

[29] Diener E, Oishi S, Lucas RE. Personality, culture, and subjective well-being: emotional and cognitive evaluations of life. Annu Rev Psychol. 2003;54:403–25. doi: 10.1146/annurev.psych.54.101601.145056 12172000

[30] Joshanloo M. Eastern Conceptualizations of Happiness: Fundamental Differences with Western Views. J Happiness Stud. 2013;15(2):475–93. doi: 10.1007/s10902-013-9431-1

[31] Uchida Y, Norasakkunkit V, Kitayama S. Cultural Constructions of Happiness: Theory and Empirical Evidence. Happiness Studies Book Series. Springer Netherlands. 2013. p. 269–80. doi: 10.1007/978-94-007-5702-8_14

[32] Al-Manji H. Development of a battery of diagnostic tests for anxiety disorders according to the Diagnostic and Statistical Manual of Mental Disorders in adults in Oman. Oman: A’Sharqiyah University. 2025.

[33] Hu L, Bentler PM. Cutoff criteria for fit indexes in covariance structure analysis: Conventional criteria versus new alternatives. Structural Equation Modeling: A Multidisciplinary Journal. 1999;6(1):1–55. doi: 10.1080/10705519909540118

[34] Kline RB. Principles and practice of structural equation modeling. 5th ed. New York, NY: Guilford Press. 2023.

[35] Muthén LK, Muthén BO. Mplus User’s Guide. 8th ed. Los Angeles, CA: Muthén & Muthén. 2017.

[36] Cheung GW, Rensvold RB. Evaluating Goodness-of-Fit Indexes for Testing Measurement Invariance. Structural Equation Modeling: A Multidisciplinary Journal. 2002;9(2):233–55. doi: 10.1207/s15328007sem0902_5

[37] Kass RE, Raftery AE. Bayes Factors. Journal of the American Statistical Association. 1995;90(430):773–95. doi: 10.1080/01621459.1995.10476572

[38] Reise SP. The invitation of bifactor models and analyses to psychometricians and clinical researchers. Multivariate Behavioral Research. 2012;47(5):667–96. doi: 10.1080/00273171.2012.715555 24049214 PMC3773879

[39] Rodriguez A, Reise SP, Haviland MG. Evaluating bifactor models: Calculating and interpreting statistical indices. Psychol Methods. 2016;21(2):137–50. doi: 10.1037/met0000045 26523435

[40] Uchida Y, Ogihara Y. Personal happiness in cultural contexts: Examining the happiness, ripples, and interdependence. Frontiers in Psychology. 2012;3:Article 338. doi: 10.3389/fpsyg.2012.00338

[41] Joshanloo M. Factor structure and measurement invariance across gender and age groups of the Mental Health Continuum–Short Form in the USA. Personality and Individual Differences. 2016;97:109–14. doi: 10.1016/j.paid.2016.03.086

[42] American Psychiatric Association. Diagnostic and Statistical Manual of Mental Disorders, Fifth Edition, Text Revision (DSM-5-TR). 2025.

[43] Aisha T. Theories of psychological well-being at work. In: Algeria, 2021.

[44] Abu Hashim SM. The structural model of relationships between psychological happiness, the Big Five personality traits, self-esteem, and social support among university students. Journal of Faculty of Education. 2010;20(81):268–350.

[45] Fouagle O. Generalized anxiety disorder and its relationship with subjective well-being among secondary school adolescents. Guelma, Algeria: University of 8 May 1945. 2023.

[46] Malone C, Wachholtz A. The Relationship of Anxiety and Depression to Subjective Well-Being in a Mainland Chinese Sample. J Relig Health. 2018;57(1):266–78. doi: 10.1007/s10943-017-0447-4 28702737 PMC5764815

[47] Noureen S, Syed LM, Ullah S, Khalid S. Social Anxiety, Social Functioning, and Psychological Well-Being in Young Adults. https://www.researchgate.net 2022.

[48] Iani L, Quinto RM, Lauriola M, Crosta ML, Pozzi G. Psychological well-being and distress in patients with generalized anxiety disorder: The roles of positive and negative functioning. PLoS One. 2019;14(11):e0225646. doi: 10.1371/journal.pone.0225646 31774860 PMC6881031

[49] Fava GA, Rafanelli C, Ottolini F, Ruini C, Cazzaro M, Grandi S. Psychological well-being and residual symptoms in remitted patients with panic disorder and agoraphobia. J Affect Disord. 2001;65(2):185–90. doi: 10.1016/s0165-0327(00)00267-6 11356243

[50] Schlechter P, Hellmann JH, Morina N. The longitudinal relationship between well-being comparisons and anxiety symptoms in the context of uncontrollability of worries and external locus of control: a two-wave study. Anxiety Stress Coping. 2024;37(5):602–14. doi: 10.1080/10615806.2024.2306530 38248916

