# Supplementary material for: Validation of the Arab Mental Health Continuum Short Form (AMHC-SF) in Oman: Psychometric properties and factorial structure
Source: PLoS One. 2026 Jul 21;21(7):e0352184. doi: 10.1371/journal.pone.0352184 (PMC13387576; doi:10.1371/journal.pone.0352184)
Supplement: S1 File — (PDF) [file pone.0352184.s001.pdf]

Mental Health Continuum–Short Form (MHC–SF)

Original version

In the past month, how often did you feel. . .						
	Never	Once or twice a month	Once a week	Two or three times a week	Almost every day	Everyday
**Emotional well-being**						
happy	0	1	2	3	4	5
interested in life	0	1	2	3	4	5
satisfied with life	0	1	2	3	4	5
**Social well-being**						
that you had something important to contribute to society	0	1	2	3	4	5
that you belonged to a community (like a social group, your neighborhood, your city)	0	1	2	3	4	5
that our society is becoming a better place for all people	0	1	2	3	4	5
that people are basically good	0	1	2	3	4	5
that the way our society works makes sense to you	0	1	2	3	4	5
**Psychological well-being**						
that you liked most parts of your personality	0	1	2	3	4	5
good at managing the responsibilities of your daily life	0	1	2	3	4	5
that you had warm and trusting relationships with others	0	1	2	3	4	5
that you had experiences that challenged you to grow and become a better person	0	1	2	3	4	5
confident to think or express your own ideas and opinions	0	1	2	3	4	5
that your life has a sense of direction or meaning to it	0	1	2	3	4	5

Source: Yeo, Z. Z., & Suárez, L. (2022). Validation of the mental health continuum–short form: The bifactor model of emotional, social, and psychological well-being. *PLOS ONE*, 17(5), Article e0268232. https://doi.org/10.1371/journal.pone.0268232
